# 

**Published:** 1908-11-15

**Authors:** 


### ANNOUNCEMENTS.

OHIO STATE DENTAL SOCIETY.—The annual meeting of this society will be held as usual in Columbus, December 1, 2 and 3, in the Great Southern Hotel. A most excellent program is prepared and all the newest and best things will be demonstrated. This is always one of the best meetings we attend. The social and professional atmosphere is admirable and we always get an inspiration from its sessions. Every one should plan to be there if possible.

INDIANA STATE BOARD OF EXAMINERS.—The next meeting of this board will be held at Indianapolis, Monday, January 11th, 1909, and continue for four days. The meetings will be held in the State House. Full information will be given on request to Dr. F. R. Henshaw, Middletown, Indiana.

EXAMINATIONS OF DENTISTS FOR ARMY POSITIONS.—While there are at the present time no vacancies, the surgeon General desires a list of eligibles from which to select. The army dentist is employed on contract at $150.00 per month, with some perquisites. He must be a graduate of a good dental college, have a good character, be between 24 and 30 years old, and pass a physical as well as a professional examination. For full particulars address "The Surgeon General, Washington, D. C.

---

Dr. A. L. Northup, of New York City, dead. On the 31st of August Dr. Northup died in Paris, France, from heart disease, at the age of seventy years. Dr. Northup was known for his high professional attainments and conduct. He was one of the strong men of New York. He was not a scientific worker but a good and constant reader of dental literature. He had one of the best libraries in the country. He was also a splendid dental society man and did much to make the old New York Odontological Society one of the strongest and best known for many years. He was a most courteous gentleman and had a large practice. He retired from practice three years ago. Dr. Remington and Dr. W. C. Herbert succeeding him.

